# Impact of Amazonian dance on speech performance in people with Parkinson’s disease

**DOI:** 10.1016/j.isci.2026.117185

**Published:** 2026-08-10

**Authors:** Raquel Arigony Prates, Carlos Cristiano Espedito Guzzo Júnior, Maria Vitória Andreazza Duarte, Alberto Muñoz Sánchez, Patrícia Pauli, Flávia Gomes Martinez, Elren Passos-Monteiro, Adolfo M. García, Aline Nogueira Haas, Constantina Theofanopoulou

**Affiliations:** 1 School of Physical Education, Physiotherapy and Dance, Federal University of Rio Grande Do Sul, Porto Alegre, RS 90690-200, Brazil; 2 Department of Sports, School of Physical Education, State University of Pará, Belém, PA 66095-490, Brazil; 3 The Rockefeller University, New York, NY 10065, USA; 4 Institute of Health Sciences, Federal University of Pará, Castanhal, PA 66050-160, Brazil; 5 Global Brain Health Institute, Trinity College Dublin, Dublin 2, Ireland/University of California, San Franciso, San Franciso, CA, USA; 6 Cognitive Neuroscience Center, Universidad de San Andrés, Buenos Aires B1644BID, Argentina; 7 Departamento de Lingüística y Literatura, Facultad de Humanidades, Universidad de Santiago de Chile, Santiago 9170022 Chile; 8 TELL Toolkit SA, Beethovenstraat, 1077 Amsterdam, the Netherlands; 9 Jameel Arts & Health Lab, New York University, New York, NY 10003, USA; 10 Emory University, Atlanta, GA 30322, USA

**Keywords:** Parkinson’s disease, dance, speech, language, cognition

## Abstract

Dance interventions improve limb motor function in Parkinson’s disease (PD), yet their impact on speech-related motor control and overall language (e.g., semantics) remains unexplored. To test this, we conducted a 12-week exploratory clinical trial comparing Amazonian dance (two groups: north and south) with a matched-physical intensity control (Nordic walking) and ran automated speech and language analysis pre- and post-intervention. The clearest corrected effect was in the dance north group, who showed improvement in prosody (main tone) compared with Nordic walking. We observed additional consistent signals (uncorrected) for prosody and semantic organization (granularity), particularly when both dance groups were pooled relative to the Nordic walking group. These findings were further supported by subject-level consistency and covariate-adjusted analyses. Our exploratory study suggests that dance may enhance speech-motor and language functions in PD, potentially through auditory-motor coupling, improved timing, and the engagement of overlapping neural substrates between dance and speech and/or language.

## Highlights

• Amazonian dance (vs. Nordic walking) improves prosody in Parkinson’s disease
• Dance (vs. Nordic walking) shows gains in semantic organization in Parkinson’s disease
• Dance may enhance speech-motor and language functions in Parkinson’s
• Effects may reflect auditory-motor coupling, timing, or shared brain pathways

## Graphical abstract

**Figure:**
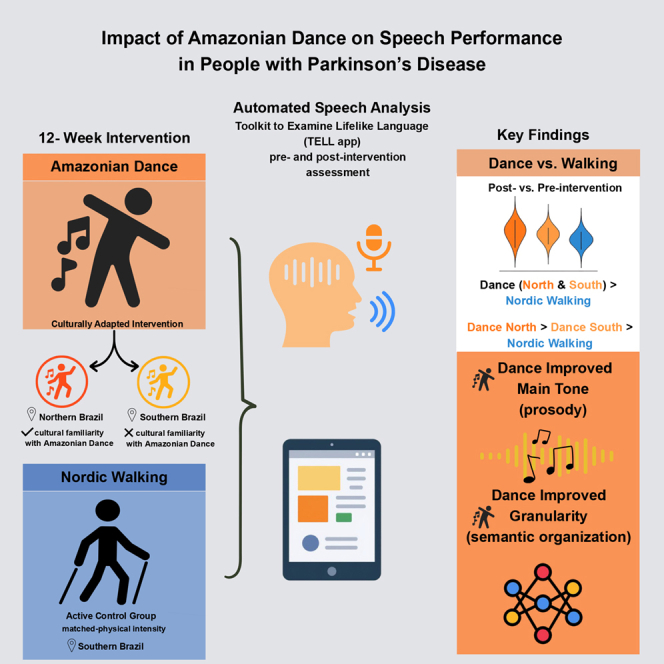

## Teaser

Health sciences; Medicine; Medical specialty; Internal medicine; Neurology; Biological sciences; Neuroscience; Cognitive neuroscience

## Introduction

Parkinson’s disease (PD) is one of the fastest-growing neurodegenerative disorders, currently affecting over 9 million people worldwide,^1^^,^^2^^,^^3^ while its prevalence continues to rise with population aging.^4^ In Brazil, a population-based study estimated a prevalence of 3.3% of PD among people older than 60.^5^ People with Parkinson’s (PwP) typically display cardinal motor impairments (e.g., resting tremor, postural instability, and bradykinesia) alongside diverse cognitive and non-motor symptoms (e.g., cognitive decline, mood disturbance, and sleep disorders).^3^^,^^6^^,^^7^ These interacting deficits collectively lead to progressive functional decline and loss of autonomy that negatively impact quality of life and well-being.

Despite substantial research on gait and limb motor function, speech remains one of the least addressed motor symptoms in PD, even though it affects up to 89% of cases.^8^^,^^9^ These deficits range from strictly motor-speech production deficits, such as reduced pitch variation, monotonous prosody, and increased pausing, to broader linguistic (non-motor) disturbances, including impoverished lexical retrieval, impaired action-verb generation, and simplified syntax.^10^^,^^11^^,^^12^ These deficits compromise intelligibility and self-expression, contributing to social withdrawal and decreased communicative confidence. Considering its reliance on finely tuned motor coordination, speech production has been proposed as a sensitive biomarker of disease progression^13^^,^^14^^,^^15^^,^^16^ with recent machine-learning approaches demonstrating high diagnostic accuracy based on acoustic and linguistic features.^17^

While various forms of speech and language therapy (SLT), such as the Lee Silverman voice treatment (LSVT), have been widely used to address PD-related speech deficits, the robustness of evidence supporting their long-term efficacy remains limited.^18^ Moreover, PwP have frequently expressed dissatisfaction with SLT methods that rely heavily on repetitive drills and isolated motor exercises, emphasizing instead the need for approaches centered on functional and communicative expressiveness.^19^^,^^20^ Another limitation lies in the demanding intensity of many SLT protocols, such as LSVT-based regimens requiring four sessions per week,^21^ which may not be feasible for many older or mobility-limited individuals. These limitations highlight the necessity of developing innovative, motivating, and ecologically valid interventions.

Dance-based interventions have emerged as promising non-pharmacological strategies for PwP, integrating rhythmic movement, social engagement, and emotional expression that may benefit both motor and non-motor domains.^22^^,^^23^^,^^24^ Numerous studies have consistently demonstrated improvements in balance, gait, and overall motor coordination following dance training.^23^^,^^24^^,^^25^^,^^26^^,^^27^^,^^28^^,^^29^^,^^30^^,^^31^^,^^32^^,^^33^ Beyond limb locomotion, dance has been proposed to engage motor brain circuits that control the laryngeal and articulatory muscles involved in speech production, such as the primary motor cortex, as well as frontal and basal ganglia circuits implicated in higher-order aspects of language production (e.g., word retrieval).^34^ In line with this hypothesis, prior studies have reported enhanced verbal fluency, word recall, and word recognition following dance-based interventions, among other improvements.^35^^,^^36^^,^^37^^,^^38^^,^^39^ However, the assessment tools used in these studies have generally lacked the granularity to disentangle whether these improvements primarily reflect changes in speech-motor control or in non-motor, language-specific processes—for instance, whether reduced word production arises from impaired memory retrieval or from difficulty in articulation—and have often relied on the subjectivity of human raters.

Building on this emerging evidence, we sought to bridge these gaps by examining whether a dance-based intervention (Amazonian dance), compared with Nordic walking, could produce measurable changes in speech-motor and/or language (non-motor) dimensions in PwP, using objective automated speech and language analysis (ASLA) features. Specifically, we employed the toolkit to examine lifelike language (TELL) app, which provides high-resolution acoustic and semantic metrics capable of parsing speech parameters that predominantly reflect motor control—such as tone, speech rate, average pause duration, and shimmer—from those indexing non-motor, language-specific processes, such as lexical-semantic precision and clustering in verbal fluency.^40^^,^^41^ Further, echoing studies demonstrating the value of culturally adapted interventions in enhancing therapeutic engagement and efficacy,^42^ the present study implemented the Amazonian dance protocol, a culturally grounded approach derived from Northern Brazilian traditions, combining rhythm, storytelling, and symbolic movement.^43^ Specifically, the intervention incorporated regional dance forms from Northern Brazil (*Lundu* and *Carimbó*), emphasizing culturally grounded themes translated into movement (e.g., representations of rivers, forests, animals, and daily life), and included participatory elements that allowed participants to engage these themes through personal and collective expression. Such culturally resonant frameworks may address the unique needs and experiences of PwP from diverse cultural backgrounds, promoting equity, reducing healthcare disparities, and ensuring culturally sensitive and patient-centered approaches that optimize quality of life. With this design, we hypothesized that participation in the Amazonian dance intervention would lead to significant improvements in selected speech and language metrics, relative to the control condition.

## Results

**Figure 1:**
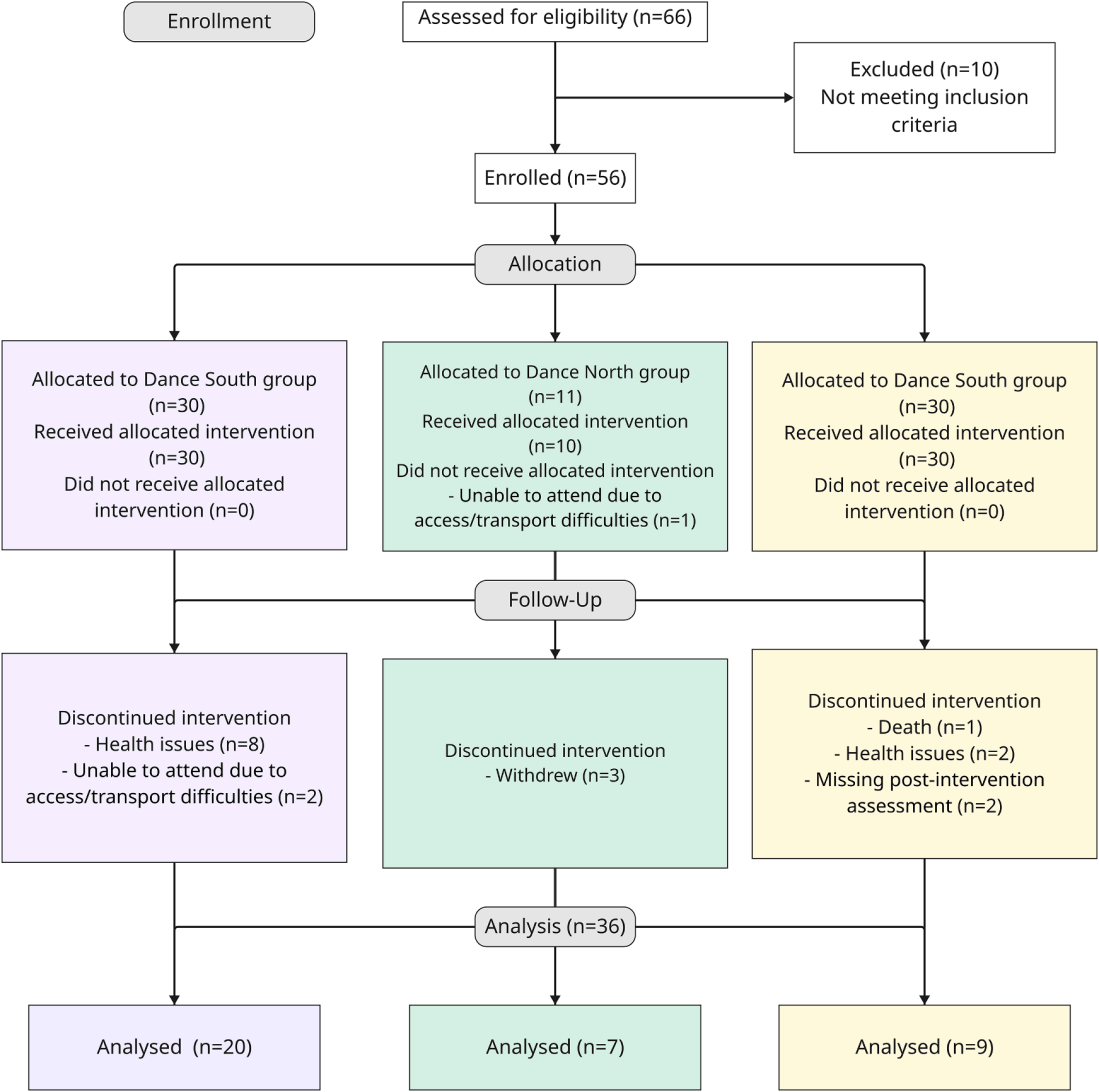
Participant flow diagram Flowchart depicting screening, randomization, intervention adherence, follow-up, and inclusion in the final analysis. Of the 66 individuals assessed for eligibility, 56 met the inclusion criteria and were assigned to one of three groups: dance south (*n* = 30), dance north (*n* = 11), or Nordic walking (*n* = 15). All participants who began the intervention were monitored throughout the 12-week program. Reasons for non-initiation or discontinuation included access difficulties, health issues, withdrawal, missing post-intervention assessments, and one death that occurred during the study period. A total of 36 participants (20 in dance south, 7 in dance north, and 9 in the Nordic walking group) completed both pre- and post-intervention speech assessments and were included in the final analyses.

The primary aim of this exploratory clinical trial was to determine whether a 12-week Amazonian dance intervention, compared with an active control condition (Nordic walking), produced measurable pre-post changes in speech-motor and/or language-related features in PwP. The Amazonian dance intervention was implemented at two sites: a dance south group (DS), comprising participants from Southern Brazil where identification with Amazonian dance traditions is less prevalent, and a dance north group (DN), comprising participants from Northern Brazil who more strongly identify with these regional cultural practices. A total of 66 individuals were screened for eligibility, of whom 56 were enrolled and assigned to three groups (dance south, dance north, and Nordic walking); attrition due to access difficulties, health issues, or incomplete post-intervention assessments resulted in a total of 36 participants with complete speech recordings: 20 participants in dance south, 7 in dance north, and 9 in the Nordic walking group (Figure 1). The final analyzed sample fell short of the *a priori* target sample size, particularly in the dance north and Nordic walking groups. In terms of demographic and clinical characteristics, at baseline, groups were broadly comparable in age and overall disease severity (Hoehn and Yahr stage), but imbalanced when it comes to sex distribution, race/ethnicity, cognitive performance, and years of education (Table 1). These imbalances, together with the reduced and uneven sample sizes, limit statistical power and the stability of subgroup-level inferences and prompted additional covariate-adjusted and subject-level analyses to assess the robustness of the findings.

**Table 1:** Participants’ demographic and clinical characteristics

	Dance south (*N* = 20)	Dance north (*N* = 7)	Dance S + N (*N* = 27)	Nordic walking (*N* = 9)
**Sex**				

Female	12 (60%)	1 (14.3%)	13 (48.1%)	5 (55.6%)
Male	8 (40%)	6 (85.7%)	14 (51.9%)	4 (44.4%)

**Ethnicity**				

Asian	1 (5%)	0 (0%)	1 (3.7%)	0 (0.0%)
Black	1 (5%)	5 (71.4%)	6 (22.2%)	1 (11.1%)
Mixed race	0 (0%)	1 (14.3%)	1 (3.7%)	0 (0%)
White	18 (90%)	1 (14.3%)	19 (70.4%)	8 (88.9%)

**Age (years)**				

Mean (SD)	71.1 (6.4)	71.6 (4.9)	71.2 (5.9)	72.6 (10.3)
Range	[57–80]	[64–78]	[57–80]	[57–86]

**H&Y**				

Mean (SD)	1.7 (0.7)	2.6 (0.5)	1.9 (0.8)	2.2 (1)
Range	[1–3]	[2–3]	[1–3]	[1–4]

**Dance experience (Gold-DSI; scale 0–7)**				

Mean (SD)	4.6 (0.8)	4.6 (0.7)	4.6 (0.8)	3.5 (0.8)
Range	[3.4–7]	[3.5–5.5]	[3.4–7]	[2–4.8]

**Education (years)**				

Mean (SD)	15 (3.5)	7 (5)	12.9 (5.2)	13.6 (6.8)
Range	[4–20]	[2–16]	[2–20]	[2–23]

**Time of diagnosis (years)**				

Mean (SD)	7.2 (5)	6.3 (2.8)	6.9 (4.5)	6 (5.2)
Range	[1–19]	[4–12]	[1–19]	[1–16]

**MoCA**				

Mean (SD)	22.6 (5.4)	17.0 (3.5)	21.1 (5.5)	20.4 (5.5)
Range	[10–30]	[11–22]	[10–30]	[11–27]

Values represent the number and percentage of participants for categorical variables and mean (standard deviation) with range for continuous variables. The table summarizes baseline characteristics for participants who completed both pre- and post-intervention speech assessments in the dance south (*n* = 20), dance north (*n* = 7), dance south + north (S + N) (*n* = 27), and Nordic walking (*n* = 9) groups. Ethnicity categories reflect self-identification using standard Brazilian census classifications. Dance experience was assessed using the Brazilian adaptation of the Goldsmiths Dance Sophistication Index (Gold-DSI; scale 0–7). MoCA scores were interpreted according to education-adjusted Brazilian cutoffs. H&Y = Hoehn and Yahr disease severity scale.

To evaluate potential attrition bias, we compared baseline characteristics (Table S1) between completers and non-completers, both across the full sample (pooled analysis; Table S2) and within each intervention group (Table S3). In the pooled sample, no significant differences were observed between completers (*n* = 36) and non-completers (*n* = 20) for age (71.6 ± 7.1 vs. 73.2 ± 8.7 years; *p* = 0.45), MoCA (20.8 ± 5.5 vs. 21.8 ± 4.6; *p* = 0.53), years of education (*p* = 0.81), Hoehn and Yahr stage (*p* = 0.73), or sex distribution (50% vs. 60% female; *p* = 0.58). Within-group analyses similarly showed no significant differences across all intervention arms (all *p* > 0.10). Overall, although attrition was substantial, these results do not indicate systematic attrition bias based on measured baseline characteristics.

Speech features were extracted from two tasks, called “animal fluency” and “routine description,” administered at baseline and post-intervention. The animal fluency task required participants to name as many animals as possible within 60 s and probed semantic memory and lexical retrieval, whereas the routine description task elicited at least 60 s of spontaneous connected speech by asking participants to describe their typical morning routine. For each task, pre-post change scores (Δ = post − pre) were computed for all acoustic and semantic metrics. Analyses were conducted at multiple levels: within-group pre-post comparisons were first performed separately for each task and subsequently for composite scores averaged across the two tasks; between-group comparisons then tested differences in change values across all group contrasts, with false discovery rate (FDR) correction applied throughout. Finally, covariate models were used to assess whether observed group effects persisted after adjustment for demographic, cognitive, and clinical factors.

### Within-group analyses per task

**Figure 2:**
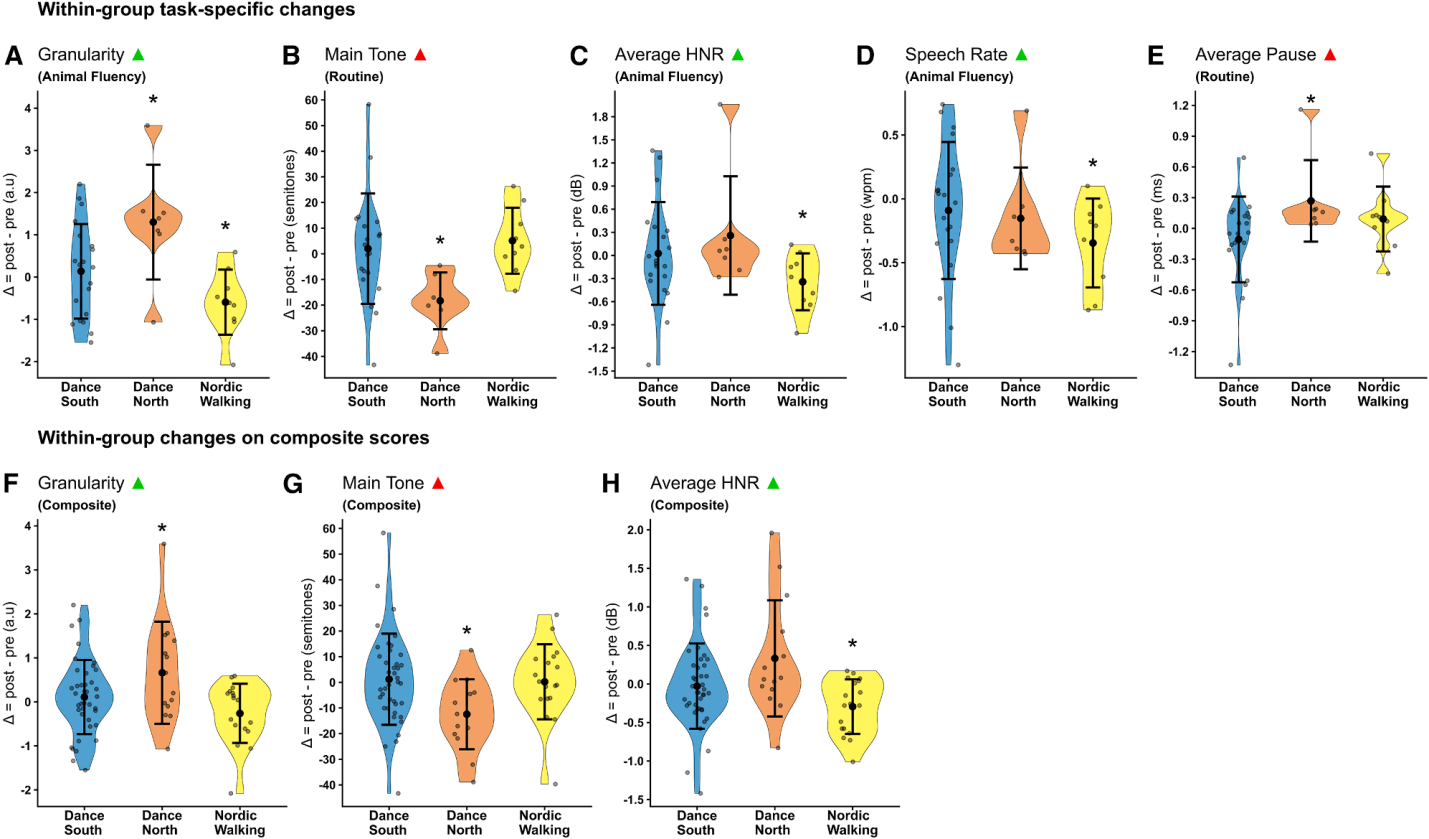
Within-group changes (Δ = post-pre) in key speech and language metrics across the three intervention arms Violin plots display the distribution of individual-level change scores for each metric, separately for dance south (blue), dance north (orange), and Nordic walking (yellow). Solid big points represent group means, the internal tick marks indicate the standard deviation, the widest part of the violin corresponds to the median, the upper and lower ends represent the first and third quartiles (Q1 and Q3), and jitter points show the raw sample size. Positive or negative Δ values reflect improvement or worsening depending on the metric’s directionality (e.g., higher Granularity and HNR indicate improvement; lower average pause duration indicates improvement). Green arrows, the higher direction indicates improvement; red arrows, the higher direction indicates deterioration. *Y* axis ranges differ across panels to reflect the natural scale of each metric (ms, semitones, etc.). Granularity is plotted in arbitrary units (a.u.), Average pause in milliseconds (ms); Speech rate in words per minute (wpm), Average HNR in decibels (dB), and main tone in semitones. Asterisks denote statistically significant within-group pre-post changes: ∗*p* < 0.05 (uncorrected). *n* values per group: dance south = 20, dance north = 7, and Nordic walking = 9.

We used within-group comparisons as an initial indication of how each intervention affected speech-motor and language features relative to participants’ own baselines (Figure 2, Tables S4 and S5). In the animal fluency task, the Nordic walking group showed three significant declines in both speech-motor and semantic features: average harmonics-to-noise ratio (HNR) decreased (Δ = −0.342 and *p* = 0.0237), indicating reduced vocal harmonicity; speech rate decreased (Δ = −0.346 and *p* = 0.0175), consistent with slower word production; and granularity decreased (Δ = −0.596 and *p* = 0.0488), reflecting less precise semantic content. In contrast, the dance north group showed a significant increase in granularity (Δ = +1.301 and *p* = 0.0446), suggesting propensity toward more fine-grained conceptual processing within the animal fluency task. No other within-group changes reached significance in this task.

In the routine task, the dance north group exhibited a large and significant decrease in main tone (Δ = −18.337 and *p* = 0.00470), a metric indexing predominant pitch, where decreases in this dataset reflect a shift toward more normative prosodic modulation. The dance north group also showed a significant change in average pause duration (Δ = +0.269 and *p* = 0.0156). Since in this feature shorter pauses signify improvement, this positive Δ reflects lengthened pauses (i.e., movement in the non-beneficial direction). No other within-group changes reached *p* < 0.05 in the routine task. None of these within-group task-level effects survived FDR at q < 0.05, although main tone in the dance north group (routine) approached that threshold (q = 0.0564). Overall, these tests enabled us to detect task-specific changes in each group before evaluating between-group differences.

### Within-group analyses on composite scores

Composite analyses, which average each speech metric across both animal fluency and routine, provide a task-independent estimate of overall pre-post change within each intervention arm (Figure 2, Tables S6 and S7). With this approach, we aimed to reduce task-specific variability and detect broad intervention-related effects. The Nordic walking group showed a significant composite decline in average HNR (Δ = −0.294 and *p* = 0.0134), consistent with worsening voice quality. The dance north group showed significant composite improvement in granularity (Δ = +0.661 and *p* = 0.0494) and a large composite decrease in main tone (Δ = −12.455 and *p* = 0.00775), again consistent with enhanced semantic output and beneficial prosodic change. A trend was observed for the Nordic walking group speech rate (Δ = −0.187 and *p* = 0.0509), suggesting a small decline in fluency that did not reach conventional significance. No composite within-group effects survived FDR at q < 0.05, although main tone (dance north) approached that level (q ≈ 0.093). Taken together, the composite analyses indicate that the most consistent within-group changes were declines in vocal harmonicity for the Nordic walking group and improvements in semantic precision and prosodic pitch for the dance north group.

### Between-group analyses per task

**Figure 3:**
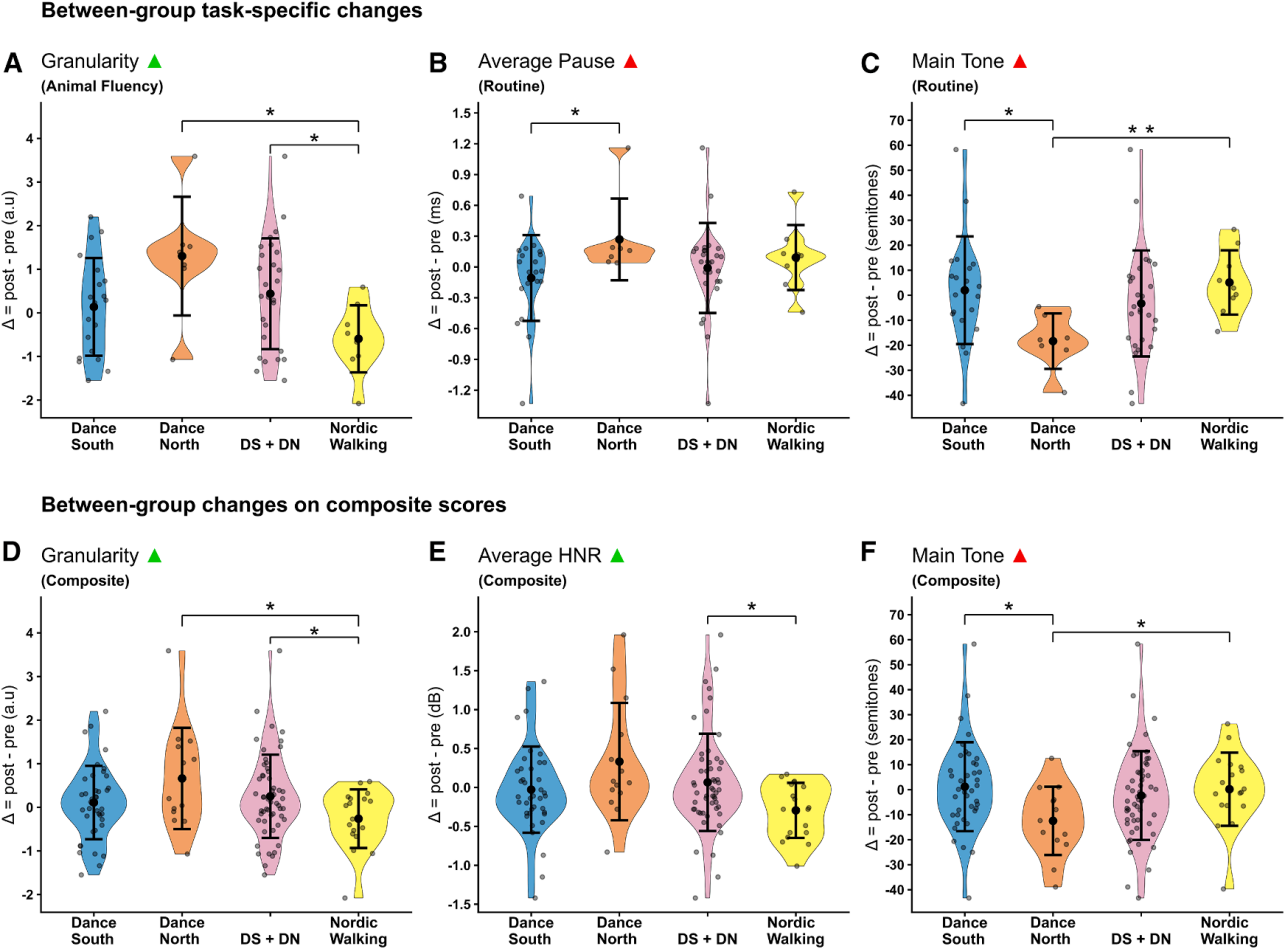
Between-group differences in pre-post change (Δ) across speech and language metrics Violin plots depict the distribution of individual Δ values (post-pre) for each intervention arm: dance south (blue), dance north (orange), pooled dance south and north (DS + DN; pink), and Nordic walking (yellow). Each panel corresponds to a metric-task combination that showed significant between-group contrasts in the statistical analyses. Positive Δ values reflect increases and negative Δ reflect decreases in the feature; whether higher or lower values indicate improvement depends on the metric (e.g., increases in Granularity or HNR reflect improvement, whereas decreases in main tone reflect improvement). Green arrows, the higher direction indicates improvement; red arrows, the higher direction indicates deterioration. Big solid points denote group means, the internal tick marks indicate the standard deviation, the widest part of the violin corresponds to the median, the upper and lower ends represent the first and third quartiles (Q1 and Q3), and jitter points show the raw sample size. Brackets indicate significant between-group contrasts, where ∗*p* < 0.05 (uncorrected) and ∗∗q < 0.05 (FDR corrected). *Y* axis ranges differ across panels to reflect the natural scale of each metric.

We then ran between-group contrasts at the task level to directly compare how each intervention influenced specific speech features relative to the other arms (Figure 3, Table S8). In animal fluency, granularity favored dance over Nordic walking in two significant contrasts: dance south + dance north vs. Nordic walking (*p* = 0.00769, *g* ≈ 0.86; mean Δ dance = +0.439 vs. Nordic walking = −0.596) and Nordic walking vs. dance north (*p* = 0.00929, *g* ≈ −1.69; Nordic walking = −0.596 vs. dance north = +1.301), indicating better content organization in the dance arms. We also noted trend-level (*p* < 0.06) contrasts, with all changes being beneficial for the dance groups, in the following tasks: average HNR (dance south + dance north vs. Nordic walking; *p* = 0.0506); granularity (dance south vs. Nordic walking; *p* = 0.0532), and average HNR (Nordic walking vs. dance north; *p* = 0.0549). All these between-group task-level effects did not survive FDR correction.

In the routine task, main tone showed the most robust between-group differences favoring the dance north group: Nordic walking vs. dance north was significant and FDR-surviving (*p* = 0.00161, q = 0.0194, and Hedges’ *g* = 1.83), with Nordic walking showing a small positive Δ (+5.11) and Dance North a large negative change (−18.34), indicating improved prosody for dance north. The contrast between dance south and dance north was also significant (*p* = 0.00448 and q = 0.0537) for this feature, with dance north showing improvement (Δ = −18.34) relative to no change in dance south (Δ = +2.00). Further in this task, Average pause duration differed between dance north and dance south (*p* = 0.0379 and Rank-biserial *r* ≈ 0.543), with dance south showing shorter pauses (Δ = −0.107) and dance north longer pauses (Δ = +0.269). Overall, the task-level analyses indicate that the strongest between-group divergences occurred in prosodic pitch (routine/main tone) and semantic detail (animal fluency/granularity), with dance groups showing movement toward lower predominant pitch and higher granularity relative to Nordic walking.

### Between-group analyses on composite scores

Similar to the within-group analyses, we then analyzed between-group comparisons in composite scores that allowed us to examine differences in overall post-pre change (Δ) across intervention arms by averaging each speech metric across both tasks, providing a broader estimate of intervention effects that is less influenced by task-specific variability (Figure 3, Table S9). On composites, the clearest differences again favored dance arms, particularly dance north, against Nordic walking. Main tone showed changes in the dance south vs. dance north (*p* = 0.00886, *g* ≈ 0.93; dance south Δ ≈ +1.21 vs. dance north Δ ≈ −12.46) and Nordic walking vs. dance north comparisons (*p* = 0.0321, *g* ≈ 1.08; Nordic walking Δ ≈ +0.21 vs. dance north Δ ≈ −12.46), reflecting greater prosodic improvements in dance north. Granularity was higher in dance vs. Nordic walking (Nordic walking vs. dance north; *p* = 0.0143, *g* ≈ −1.50; dance south + dance north vs. Nordic walking, *p* = 0.0198, *g* ≈ 0.78), demonstrating more fine-grained semantic output for the dance arms. Average HNR (dance south + dance north vs. Nordic walking) also favored the combined dance groups over Nordic walking (*p* = 0.0198; dance Δ ≈ +0.065 vs. Nordic walking Δ ≈ −0.262), consistent with better average voice quality after dance. None of these composite between-group effects survived FDR at q < 0.05, but the pattern was internally consistent with the task-level results and with the within-group changes. Overall, the composite analyses reinforce that the largest between-group differences were observed in prosodic pitch, semantic detail, and vocal harmonicity, with the dance groups, particularly dance north, showing more favorable change than Nordic walking.

### Subject-level consistency of between-group effects

To assess whether nominally significant between-group differences were supported at the participant level despite limited group sizes, we conducted a subject-level consistency analysis restricted to contrasts with *p* < 0.05 in the primary between-group analyses (Table S10). We found that comparisons between dance north and Nordic walking showed the clearest and most consistent separation between groups. For routine/main tone, all participants in the dance north group (7/7) showed change in the beneficial direction, compared with a minority of participants in Nordic walking (3/9), which yielded a significant difference in the proportion of beneficial responders (two-sided Fisher’s exact, *p* = 0.0114). We observed a similar pattern for animal fluency/granularity, where a higher proportion of participants in dance north (6/7) showed beneficial change relative to Nordic walking (2/9) (*p* = 0.0406). These and the rest of task-level findings confirmed that the primary between-group differences for these features were not driven by a small subset of participants but reflect consistent directional changes across individuals.

Analyses on composite scores showed partially convergent results. We again found that, for main tone, the dance north group exhibited a higher proportion of beneficial responders than Nordic walking (7/7 vs. 4/9; *p* = 0.0337), consistent with the task-level findings. For Granularity, we also observed a higher proportion of beneficial responders in dance north relative to Nordic walking (6/7 vs. 2/9; *p* = 0.0406), although this pattern was less consistent across other between-group contrasts. Other nominally significant between-group effects, including those involving Average HNR or pooled dance-versus-walking comparisons, showed weaker patterns at the participant level. Taken together, these results indicate that the most robust between-group differences identified in the primary analyses, particularly those involving dance north relative to Nordic walking, are supported by consistent directional changes across participants, whereas more marginal effects are less stable.

### Covariate analyses

To assess whether the observed group differences in the most robust speech outcomes could be explained by key clinical characteristics, we ran multivariable ANCOVA models including group, age, H&Y, and MoCA for the four speech features that survived both the primary between-group analyses and the subject-level consistency analyses: animal fluency/granularity, routine/main tone, composite/granularity, and composite/main tone (Tables S11, S12, and S13). Even after this adjustment, group remained a significant predictor for all four outcomes, confirming that the principal between-group effects are not reducible to baseline differences in age, disease severity, or cognitive status. In animal fluency, group remained significant for granularity (*p* = 0.0068, q = 0.0272, and partial η^2^ = 0.283). In routine, group remained significant for main tone (*p* = 0.0176, q = 0.0273, and partial η^2^ = 0.236). At the composite level, group also remained significant for granularity (*p* = 0.0205, q = 0.0273, and partial η^2^ = 0.228) and main tone (*p* = 0.0327, q = 0.0327, and partial η^2^ = 0.204). Among the covariates entered in these same models, H&Y showed significant associations with granularity in both animal fluency (*p* = 0.0189, q = 0.0378, and partial η^2^ = 0.170) and the composite analysis (*p* = 0.0187, q = 0.0378, and partial η^2^ = 0.171), whereas age and MoCA were not significant for any of the retained outcomes. Thus, the clearest semantic and prosodic between-group differences remained robust after covariate adjustment, while disease stage contributed specifically to variability in semantic change.

We then used single-covariate models to test whether each covariate (age, H&Y, or MoCA) showed any systematic associations with these same outcomes when entered with group one at a time. This analysis confirmed the same overall pattern. H&Y was significantly associated with granularity in animal fluency (*p* = 0.0093, q = 0.0201, and partial η^2^ = 0.193) and at the composite level (*p* = 0.0101, q = 0.0201, and partial η^2^ = 0.190), whereas age and MoCA were not significantly associated with any of the retained outcomes. The simple linear regression analyses showed the same directionality: higher H&Y scores were associated with larger positive Δ values for granularity in both animal fluency (β = 0.654, r = 0.439, and *p* = 0.0075) and the composite analysis (β = 0.351, r = 0.432, and *p* = 0.0086). Since higher Granularity is interpreted as beneficial, this pattern suggests that participants with more advanced disease stage tended to show larger semantic gains overall. Taken together, these findings indicate that disease severity contributed to inter-individual variability in semantic change, but the principal group-level effects for granularity and main tone remained significant after adjustment and therefore cannot be explained solely by baseline differences in age, cognition, or disease stage.

## Discussion

**Figure 4:**
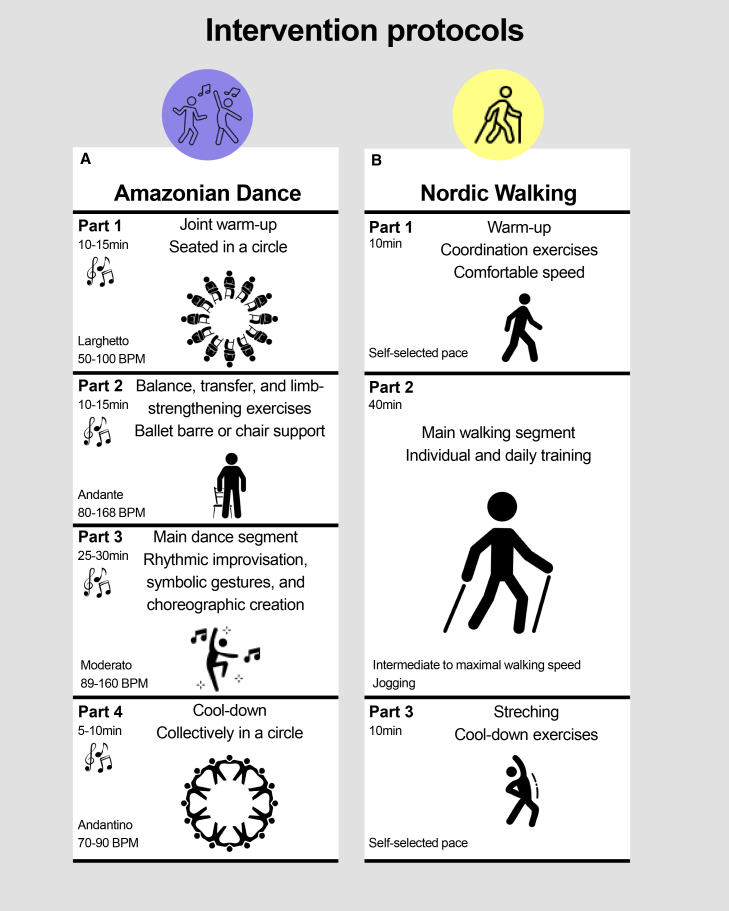
Structure of the Amazonian dance and Nordic walking intervention protocols (A) Amazonian dance protocol*.* Each 60-min session consisted of four sequential parts: Part 1 (10–15 min)—joint warm-up performed seated in a circle; part 2 (10–15 min)—balance, transfer, and limb-strengthening exercises performed with support from a ballet barre or chair; part 3 (25–30 min)—main dance segment incorporating rhythmic improvisation, symbolic gestures, and choreographic creation; and Part 4 (5–10 min)—cool-down performed collectively in a circle. Musical tempo varied across parts, progressing from *Larghetto* (50–100 BPM) to *Andante* (80–168 BPM), *Moderato* (89–160 BPM), and *Andantino* (70–90 BPM). The protocol was culturally adapted and standardized for both the Northern and Southern Brazilian sites. (B) Nordic walking protocol. Each 60-min session consisted of three parts: part 1 (10 min)—warm-up and coordination exercises at a self-selected pace; part 2 (40 min)—main walking segment performed at intermediate to maximal walking speed or light jogging as tolerated; and part 3 (10 min)—stretching and cool-down exercises at a self-selected pace.

This exploratory clinical trial aimed to assess the impact of an Amazonian dance protocol on speech and language metrics in Brazilian PwP from two different Brazilian regions (north and south), compared with a time- and dose-matched Nordic walking intervention (Figure 4). The clearest corrected between-group effect was observed for prosodic control (main tone), particularly in the contrast between dance north and Nordic walking. In addition, we observed consistent but uncorrected signals for prosody (main tone) and semantic organization (Granularity), including when both dance groups were pooled relative to Nordic walking. Importantly, these effects were supported by subject-level consistency analyses, which showed a higher proportion of participants exhibiting beneficial change in the dance groups for prosody and semantic organization, indicating that the observed group differences were reflected in consistent directional changes across individuals. Even after covariate analysis, the significant effect of dance interventions on prosodic and semantic features remained robust, suggesting that these findings are not fully explained by baseline differences in disease severity, cognition, or age. At the same time, disease stage (H&Y) contributed to inter-individual variability in semantic outcomes, indicating that baseline clinical status modulates the magnitude of change. These findings suggest that dance interventions may influence selected prosodic and semantic features in PwP; however, given the limited sample size, differential attrition, baseline group imbalances, and the fact that most effects did not survive correction for multiple comparisons, the results should be interpreted as exploratory and hypothesis-generating rather than confirmatory.

Although dance interventions are well established for motor and psychosocial benefits in PD^44^^,^^45^^,^^46^^,^^47^ direct evidence for speech metrics has been scarce. The most directly comparable study is Karimi et al.,^48^ who reported improvement in pitch stability in PwP after 5 years of weekly dance classes—consistent with our prosody improvements in the 12-week protocol. Other studies summarized in Theofanopoulou^34^ have documented gains in verbal fluency, lexical retrieval, and semantic clustering after dance training, though none clarified whether these reflected speech-motor or language-semantic processes. Our study results complement and extend this evidence, showing improvements in both prosodic control (main tone) and semantic precision (granularity), quantified using automated speech and language analysis. This dual improvement suggests that dance can influence multiple layers of communication—from motor execution to lexical-semantic organization.

The more pronounced gains in the Amazonian dance north group should be interpreted cautiously. While these patterns may be consistent with greater cultural familiarity with Amazonian dance forms in Northern Brazil, this factor was not directly measured. However, we highlight the importance of cultural embodiment and regional identity. Participants’ familiarity with *Lundu* and *Carimbó*, traditional Northern Brazilian dances, may have amplified emotional engagement and motivation, fostering a stronger sense of belonging and expressive agency. Cultural embodiment may enhance adherence, self-expression, and social reward responses, all of which may facilitate deeper involvement in rhythmic, socially rich activities.^34^^,^^49^ This finding aligns with recent meta-analyses emphasizing that culturally adapted and group-based performing arts interventions improve affective engagement and adherence.^50^^,^^51^ In this context, Amazonian dance may act as both a motor training and a sociocultural rehabilitation practice, engaging identity, and emotional resonance as mediators of communicative vitality.

The observed improvements are also consistent with emerging mechanistic hypotheses regarding how dance influences speech-related brain pathways. Theofanopoulou^34^ proposed that dance training may affect not only limb-motor circuits but also the neural pathways controlling laryngeal and orofacial movements, supported by evidence that single neurons in the primary motor cortex can control both limb and vocal-motor actions. Neuroimaging studies across healthy adults and individuals with PD or MCI have shown dance-related changes in basal ganglia-premotor connectivity, frontal and temporal cortical plasticity, precentral gyrus structure, and networks spanning auditory, limbic, and sensorimotor areas.^52^^,^^53^^,^^54^^,^^55^^,^^56^^,^^57^^,^^58^ Such findings suggest that dance can modulate distributed neural circuits relevant not only speech-motor (e.g., prosody and timing) but also to non-motor/language (e.g., semantic organization) production.

The Nordic walking group, by contrast, demonstrated declines in granularity, HNR and speech rate, over the intervention period. These changes may reflect multiple, non-mutually exclusive factors. One possibility is disease-related change over the 12-week period, including potential disease progression. In PwP, such changes are consistent with well-characterized alterations in speech production, including reduced voice quality, altered rhythm, and prosodic flattening.^59^^,^^60^ A second possibility is limited transfer of general aerobic exercise to speech-motor or language-semantic domains, which may relate to differences in how the two interventions engage speech-relevant processes. Although Nordic walking is effective for improving gait and balance, it does not incorporate expressive and music-based components^44^^,^^61^ in the way dance does. This may be because although Nordic walking involves intrinsic rhythmicity, it does not rely on externally cued, music-based entrainment that is predictive, flexible, and continuously adaptive, all of which are key characteristics of the rhythmicity that takes place when dancing. The latter includes anticipating beat timing, aligning movement with hierarchical musical structure (e.g., phrasing), and dynamically adjusting to tempo variations, in contrast to the rhythmicity^62^ akin to Nordic walking, which is largely self-generated and less dependent on continuous auditory prediction and adaptation. Short-term variability in performance may also contribute to these patterns, although this is unlikely to fully account for the observed directional changes. These interpretations strengthen the notion that dance interventions may tap into neural systems beyond those targeted by conventional physical activity, yielding more direct benefits for speech and language.

In our covariate-adjusted analyses, the principal between-group effects for granularity and main tone remained significant, indicating that these differences are not fully explained by baseline variation in age, cognition, or disease stage. Among the covariates, disease severity (H&Y) was selectively associated with Granularity, showing that clinical severity contributes to variability in semantic change, whereas age and MoCA were not significant predictors. We interpret this as indicating that the observed group effects persist beyond baseline clinical differences, while also suggesting that disease severity modulates the magnitude of semantic gains. More broadly, this pattern points to inter-individual variability in responsiveness—particularly for semantic organization—and may inform future study designs targeting PwP at later disease stages.

In conclusion, this study provides preliminary, exploratory evidence that a culturally adapted Amazonian dance protocol can meaningfully modulate speech-motor and linguistic-semantics metrics in PwP. By combining automated speech and language analysis with a standardized 12-week intervention and a physically active control group, this work offers a reproducible framework for quantifying communication outcomes in dance research. The results highlight consistent signals for motor control and non-motor aspects of speech/language, but they should still be interpreted cautiously given the reduced sample size, group imbalances, and attrition. These findings should be considered hypothesis-generating and require confirmation in larger, adequately powered randomized trials. Overall, this study offers a promising direction for integrative, culturally sensitive rehabilitation in PD.

### Limitations of the study

A major limitation of this study is that the final sample was substantially smaller than the sample size planned in the *a priori* power calculation, particularly in the dance north and Nordic walking groups. Attrition resulted in reduced group sizes, especially in Dance North and Nordic walking, limiting statistical power, reducing the precision and stability of subgroup and covariate analyses, and decreasing the strength of the inferences that can be drawn from the results. In addition, baseline differences across groups further complicated attribution of observed effects to the intervention alone. Although groups were broadly comparable in age and overall disease severity (H&Y), and the strongest effects remained significant after covariate adjustment, all findings should be interpreted cautiously and as exploratory. Another limitation is its reliance on a single pre- and post-intervention assessment per participant. Given the known day-to-day variability in PD symptoms, repeated baseline assessments would have provided a more stable reference point and improved the reliability of estimated change scores. In addition, the pre-post design without follow-up restricts the ability to draw conclusions regarding long-term retention or delayed intervention effects. Future trials should incorporate longitudinal follow-up assessments, including perceptual and patient-reported communication outcomes, to validate and expand upon these preliminary findings. Another limitation concerns the relatively narrow set of speech tasks and acoustic features employed. This restricted battery may not fully capture the complexity and heterogeneity of speech and language profiles in PwP. Future research should incorporate a broader range of ecologically valid tasks and more comprehensive linguistic-acoustic measures to enhance the characterization of dance-related changes in speech and language.

## Resource availability

### Lead contact

Requests for further information should be directed to and will be fulfilled by the lead contact, Constantina Theofanopoulou (ktheofanop@rockefeller.edu).

### Materials availability

This study did not generate new unique reagents or materials.

### Data and code availability

• Coded raw numerical data corresponding to TELL features and analysis data have been shared in supplementary tables. Fully raw data (i.e., voice recordings) cannot be shared because voice is personally identifying information.
• No custom code or software was generated or used in this study.
• Any additional information required to reanalyze the data reported in this paper is available from Aline Nogueira Haas (alinehaas@gbhi.org) and Constantina Theofanopoulou (ktheofanop@rockefeller.edu) upon request.

## Acknowledgments

The authors acknowledge the participants in the study for their invaluable collaboration.

This research was supported by the Alzheimer’s Association in partnership with the 10.13039/100015442Global Brain Health Institute (GBHI) under grant application GBHI ALZ UK-24-1068625 and in part by the Coordenação de Aperfeiçoamento de Pessoal de Nível Superior – Brasil (10.13039/501100002322CAPES) – Finance Code 001. Constantina Theofanopoulou acknowledges funds from The Rockefeller University and New York University (Jameel Arts and Health Lab). Adolfo M. García is partially supported by the National Institute On Aging of the National Institutes of Health (R01AG075775 and 2P01AG019724); ANID (FONDECYT Regular 1250317, 1250091); 10.13039/501100003074Agencia Nacional de Promoción Científica y Tecnológica (01-PICTE-2022-05-00103); and the Multi-partner Consortium to Expand Dementia Research in Latin America (ReDLat), which is supported by the 10.13039/100000061Fogarty International Center at the National Institutes of Health, the 10.13039/100000049National Institute on Aging (R01AG057234, R01AG075775, R01AG21051, and CARDS-NIH), 10.13039/100000957Alzheimer's Association (SG-20-725707), Rainwater Charitable Foundation’s Tau Consortium, the Bluefield Project to Cure Frontotemporal Dementia, and the 10.13039/100015442Global Brain Health Institute.

## Author contributions

Conceptualization, R.A.P., C.T., and A.N.H.; data curation, R.A.P., C.T., C.C.E.G.J., M.V.A., and P.P.; formal analysis, C.T., R.A.P., M.V.A.D., A.M.S., and P.P.; funding acquisition, R.A.P., A.N.H., and C.T.; supervision, A.N.H. and C.T.; writing – original draft preparation, R.A.P., C.T., and A.N.H.; writing – review and editing, R.A.P., C.C.E.G.J., M.V.A.D., A.M.S., F.G.M., E.P.-M., A.M.G., A.N.H., and C.T. All authors read and approved the final manuscript.

## Declaration of interests

The authors declare no competing interests.

## Declaration of generative AI and AI-assisted technologies in the writing process

During the preparation of this work, the authors used ChatGPT (OpenAI) to assist with sentence rephrasing, grammar and spelling checks, and stylistic refinement, particularly to support clarity for authors who are not native English speakers. After using this tool or service, the authors reviewed and edited the content as needed and took full responsibility for the content of the publication.

## STAR★Methods

### Key resources table

**Table:** 

REAGENT or RESOURCE	SOURCE	IDENTIFIER
**Deposited data**		

Data for the “Impact of Amazonian Dance on Speech Performance in People with Parkinson’s Disease” trial	https://data.mendeley.com/datasets/3wgxpsbch7/1	https://doi.org/10.17632/3wgxpsbch7.1

**Software and algorithms**		

Microsoft Excel	Microsoft Corporation	N/A
Adobe Illustrator 2020	Adobe	N/A

**Other**		

Human participants	People with Parkinson’s disease (PwP)	Diagnosed by neurologist, H&Y I–III
Unified Parkinson’s Disease Rating Scale (UPDRS)	Movement Disorder Society	https://www.movementdisorders.org

### Experimental model and study participant details

This study followed an exploratory clinical trial with a parallel-group design, approved by the Hospital de Clínicas de Porto Alegre (HCPA) Research Ethics Committee (CAAE: 75675023.9.0000.5327) and by the Federal University of Pará Ethics Committee (CAAE: 75675023.9.3002.0018). All the participants provided written informed consent prior to enrollment.

Sample size was determined following the protocol of a previously published study^24^ using GPower 3.1 software, based on an Analysis of Variance (ANOVA) - within/between interaction model (effect size = 0.40, α = 0.05, power = 0.95). The analysis indicated that a minimum of 16 individuals per group would be required. Allowing for an estimated 20% attrition rate, we targeted for enrollment of 20 individuals per group.

Participants were recruited from Dance and Nordic Walking Community Projects for PwP, Parkinson’s Associations, via social media and flyers distributed in two different regions of Brazil (North and South). Inclusion criteria were: a diagnosis of idiopathic PD, based on standard clinical diagnostic criteria determined by a neurologist^63^; age 50-80 years; both sexes; social-economically and racially diverse backgrounds; Hoehn and Yahr (H&Y) stages I-III; ≥ 1 year of stable medical treatment for PD with regular use of anti-parkinsonian drugs; ability to understand the verbal instructions for the tests and to walk with no walking aid; and native proficiency in Brazilian Portuguese with normal or corrected-to-normal vision and hearing. Exclusion criteria included the presence of other associated neurological or chronic diseases, recent surgery, or signs of Parkinson-plus syndromes.

The participants were allocated into three groups based on study site and practical considerations related to intervention delivery. In this process, age and disease severity (Hoehn & Yahr) were considered to support overall comparability across groups, although no formal randomization procedure was implemented. Two groups received the Amazonian Dance intervention, delivered over 12 weeks, with two sessions per week, each lasting one hour. Dance-South Group (DS) comprised participants from Southern Brazil, where identification with Amazonian Dance is less prevalent, whereas Dance North Group (DN) comprised participants from Northern Brazil, who strongly identify with these regional cultural practices. The active control group, Nordic Walking Group (NW) completed a 12-week Nordic Walking intervention, also twice a week, with two one- hour sessions per week.

### Method details

#### Amazonian dance and nordic walking protocol

The instructor-led Amazonian dance and Nordic walking protocol were specifically designed for PwP, carried out in accessible and safe spaces and led by qualified instructors experienced in Parkinson’s population. The Amazonian Dance protocol was standardized and applied identically in both the South and North regions of Brazil. All segments of the Amazonian Dance protocol (Figure 4) were performed to music and were conceived as part of the dance practice, including warm-up elements, such as balance or strength-focused movements, which were embedded within a rhythmic, dance-based framework rather than constituting separate exercise modalities. Nordic Walking was chosen as an active control condition because it is a well-established, low-cost, safe, and effective rehabilitation strategy for PwP.^64^ It is also based on a multi-limb physical activity with comparable intensity, duration, and social structure when compared to the dance protocol. While both interventions involve coordinated movement and rhythmic structure, the hypothesized distinction lies in the externally cued, music-based rhythmic entrainment, expressive movement, and enhanced auditory-motor integration inherent to dance. The dose of both interventions—12 weeks, twice a week, 2 hours per week—was determined based on previous studies demonstrating optimal engagement and feasibility for older adults and PwP.^65^^,^^66^ Detailed descriptions of the Amazonian Dance protocol^43^ and the Nordic Walking protocol^67^ have been published elsewhere.

#### Instruments and data collection procedures

All participants attended two data-collection sessions: a baseline evaluation (pre-intervention) and a post-intervention evaluation, after the 12-week program. To minimize motor-state fluctuations, all assessments were conducted during the medication “ON” state, between one to three hours after the participant’s regular dose of anti-parkinsonian medication. Pre- and post-intervention measurements were assessed at the same time of the day, while data collectors were blinded to the participants’ groups. Assessments were carried out by trained, experienced and qualified healthcare professionals.

Demographic and clinical data (age, sex, ethnicity, years of education, and years since the diagnosis) were collected using a structured anamnesis tool. The Brazilian version of the Goldsmith’s Dance Sophistication Index (Gold-DSI) was used to assess the participants’ dance experience.^68^ The Gold-DSI comprises 26 items assessing individual experiences of performing, watching, and knowing about dance. A Likert scale is used to classify the scores: not at all = 0; beginner = 1 to 2, intermediate = 3 to 4, advanced = 5 to 6, professional = 7. Global cognition was assessed using the Montreal Cognitive Assessment (MoCA), applying Brazilian education-adjusted cutoffs for mild cognitive impairment (MCI) and dementia^69^: level of education: ≥ 12 years – MCI (mild cognitive impairment) ≤ 22 score, dementia ≤ 21.5 score; 9-11 years - MCI ≤19.5 score, dementia ≤ 19.5 score; 5-8 years - MCI ≤ 19.5 score, dementia ≤ 16.5 score; 1-4 years - MCI ≤ 18 score, dementia ≤ 15.5 score; 0 years - ≤ 11.5 score, dementia ≤ 8.5 score. Disease stage was determined by the Hoehn and Yahr (H&Y) scale,^70^ ranging from Stage I (unilateral involvement) to V (wheelchair-bound or bedridden), with higher stages indicating more advanced motor impairment.

#### Speech data acquisition, processing and feature extraction

All speech and language data were collected and analyzed using the Toolkit to Examine Lifelike Language (TELL), a validated web-based platform that extracts acoustic, prosodic, and linguistic biomarkers sensitive to neurodegenerative disease. TELL provides automated, high-precision speech and language metrics based on naturalistic spoken responses.^40^^,^^41^

Participants completed two standardized speech tasks within the TELL app environment: 1) Animal Fluency: This is a widely used clinical measure of semantic memory and lexical retrieval. Participants were given 60 seconds to name as many different animal names as possible without repetition or derivational variants. The instructions for this task were as follows: ‘Name as many animals as you can in one minute. You may not repeat items, use proper names, or use words derived from the same root (e.g., table, tables, little table).” If the participant stopped speaking before the end of the minute, they were prompted with: “Can you think of more?”; 2) Routine Description: This task elicits spontaneous narrative speech for at least 60 seconds, requiring participants to describe their typical morning routine in detail. The instructions for this task were as follows: ‘Now tell me what a typical morning in your life is like. Give me as many details as you can. For example, instead of saying “I make breakfast” tell me everything you do to prepare breakfast. If the participant did not speak for at least one minute, we prompted them with: “Tell me more.”

All recordings were conducted individually with only the examiner and the examinee present, in a quiet room free of external noise. Participants were instructed to sit comfortably, silence electronic devices, remove jewelry that could interfere acoustically, and maintain a fixed distance from the microphone. An Apple iPad Air was used for speech recording, using its built-in microphone. Audio files were recorded and saved as single-channel Wave (.wav) files, with a sampling rate of 44.1 kHz and a bit-depth of 24 bits. Data collection was administered by trained healthcare professionals blinded to group assignment. Speech data were processed using the TELL automated pipeline, which integrates multiple preprocessing, segmentation, and feature extraction steps.^15^^,^^40^^,^^41^^,^^71^ To improve reproducibility, the full workflow is described below.

Audio preprocessing: Following a validated pipeline, audio files were downmixed to mono, resampled to 16 kHz, and bandpass-filtered (200–3,400 Hz) to retain the speech-relevant frequency range. Signals were then loudness-normalized using the FFmpeg multimedia framework and subjected to background noise reduction using the FullSubNet model. Speech and non-speech segments were identified using the Silero voice activity detection model. Recordings were subsequently diarized using pyannote to identify and remove segments containing examiner speech.

Transcription, alignment, and preprocessing: Audio recordings were transcribed using Whisper (v3.0) and manually reviewed by two trained annotators following standardized criteria. Speech segments were further processed to derive inter-pause units and were force-aligned to words, syllables, and phonemes using forced-alignment tools (e.g., FASEAlign). Transcripts were then lemmatized and/or tokenized depending on downstream analysis requirements, and structured datasets (.csv files) were generated for statistical analysis.

Speech timing features: Speech timing features were extracted using PRAAT scripts. These included number of syllables (identified via intensity peaks), number of pauses (silent intervals), mean syllable duration, mean pause duration, and articulation rate (number of syllables per unit of speech time excluding pauses). Mean values for each feature were computed per recording.

Pitch and prosodic features: Prosodic features were derived from analysis of the fundamental frequency (F0) contour and energy contour across each recording. Specifically, we quantified the slope of the interpolated F0 contour and the slope of energy changes within voiced frequency bands across the narration, yielding subject-level measures of pitch and prosodic modulation.

The analyses focused on ten speech metrics, all shown to be sensitive to PD^16^^,^^71^^,^^72^: 1) Speech rate refers to the speed of speech production and is quantified in syllables per unit of time. Very high values may be associated with disinhibition, fluent aphasia, or manic episodes, whereas low values may indicate specific patterns of dysarthria or apraxia of speech, which are common in PD^73^^,^^74^^,^^75^; 2) Average pause duration captures the mean length of silences between sentences, words, or parts of words. Longer-than-normal pauses have been observed in conditions such as PD, AD, nonfluent primary progressive aphasia, depression, and schizophrenia, while sudden short pauses may be related to hypomanic states^76^^,^^77^^,^^78^; 3) Average syllable duration reflects the mean length of syllables in speech, with higher values suggesting motor speech difficulties commonly seen in PD, apraxia of speech, traumatic brain injury, and mild cognitive impairment^72^^,^^77^^,^^79^^,^^80^; 4) Pause duration variability captures fluctuations in the duration of pauses and may indicate impaired speech rhythm in conditions including PD, frontotemporal dementia, progressive nonfluent aphasia, amyotrophic lateral sclerosis, AD, depression, schizophrenia, and amnestic mild cognitive impairment^76^^,^^77^^,^^78^; 5) Syllable duration variability reflects variation in syllable length, and tends to be reduced in parkinsonian, ataxic, or right-hemisphere dysarthria, whereas increased variability has been reported in hypokinetic dysarthria^81^^,^^82^^,^^83^; 6) Harmonic-to-noise ratio (HNR) measures vocal efficiency by assessing the proportion of harmonic energy relative to noise, with low values suggesting asthenic voice or dysphonia, both being pathological voice conditions common in PD^84^^,^^85^; 7) Shimmer indexes cycle-to-cycle variability in amplitude, with increased values observed in PD and AD, reflecting irregular vocal fold vibration^86^^,^^87^; 8) Main tone refers to the predominant vocal pitch across the sample. In Parkinson’s disease, reduced pitch variability (monopitch) is a core feature of hypokinetic dysarthria. In the present analyses, improvement is operationalized as movement away from abnormally elevated or flattened pitch patterns toward a more normative pitch range within the task context.^74^^,^^88^^,^^89^ Accordingly, decreases in Main Tone in this dataset reflect a shift toward more appropriate prosodic modulation rather than a simple lowering of pitch per se; 9) Semantic granularity reflects the degree of semantic specificity and content richness of the words produced, ranging from broad and unspecific terms (e.g., “entity,” “thing”) to more fine-grained categories (e.g., “animal,” “dog,” “bulldog”). Low values may indicate impaired access to semantic memory^72^^,^^90^^,^^91^; 10) Ongoing semantic variability (OSV) quantifies the semantic distance between consecutive words and examines the variability of these distances across the narrative. High variability suggests flexible semantic exploration, whereas low variability indicates restricted or stereotyped semantic production.^90^^,^^91^ Together, the first eight features primarily index speech-motor and phonatory control, capturing articulatory timing, prosodic rhythm, and vocal stability; in contrast, the final two features—semantic granularity and ongoing semantic variability—reflect higher-order language and semantic processing, providing complementary information about lexical access, semantic organization, and discourse structure.

### Quantification and statistical analysis

Descriptive statistics were first computed for all demographic and clinical variables (comparing baseline group, including completers and non-completers participants) and are reported as means, standard deviations, medians (interquartile ranges), and proportions. Continuous variables were assessed for normality using the Shapiro–Wilk test. Normally distributed variables (age and MoCA scores) were summarized as mean ± standard deviation and compared using independent samples t-tests. Non-normally distributed variables (years of education and Hoehn & Yahr stage) were summarized as median (interquartile range) and compared using Mann–Whitney U tests. Categorical variables (sex) were summarized as counts and percentages and compared using Fisher’s exact tests. Pre- and post-intervention data from two speech tasks, Animal Fluency and Routine, were analyzed to examine changes in speech performance across three groups: Dance South (DS), Dance North (DN) and Nordic walking (NW). Each participant contributed data at two timepoints (pre- and post-intervention), and for each speech metric individual-level change score (Δ = post − pre) was computed. Positive Δ values indicated increases and negative values indicated decreases in the measured metric. Importantly, whether a positive Δ reflected functional improvement depended on the nature of the metric: for example, higher speech rate or higher Harmonics-to-Noise Ratio (HNR) are considered improvements (so positive Δ is beneficial), whereas lower average pause duration and lower shimmer variability are considered improvements (so negative Δ is beneficial). These directional expectations were established *a priori* based on the known psychometric properties of each feature and are summarized in the TELL Speech Feature Codebook (DATA S1/Methods S1).

To run analysis at multiple levels, the data was first organized in a long-format within-subject table containing, for each participant, task, and metric, their pre, post, and Δ values (Table S4). Because each metric was collected separately during the Animal Fluency and Routine tasks, in addition to individual task analyses, a cross-task composite value for each metric by averaging the scores from the two tasks for each participant was computed, producing an individual-level table of composite pre, post, and Δ values (Table S6). These individual-level tables served as the basis for all subsequent group-level analyses.

Within each group, pre-post changes were tested using paired-samples t-tests when normality assumptions were met, and Wilcoxon signed-rank tests otherwise. These analyses were performed both at the task level (separately for Animal Fluency and Routine) (Table S5) and at the composite level (combining the two tasks) (Table S7). For each group × task × metric combination, the number of paired observations were reported, pre and post means with 95% confidence intervals (CIs), the mean Δ and its 95% CI, the test statistic, the *p*-value, and the effect size as Cohen’s d (for t-tests).

To compare the magnitude of change across groups, between-group analyses were conducted on the individual Δ scores. These tests assessed all pairwise group contrasts (DS vs NW, NW vs DN, DS vs DN) as well as a combined contrast comparing DN+DS (the two dancing groups) versus NW (the walking group). Analyses were performed separately for the two tasks (Table S8) and for the composite scores (Table S9). Welch’s t-tests were applied when normality assumptions were met, and Mann-Whitney U tests otherwise. For each contrast, the group means of Δ with 95% CIs, the test statistic, uncorrected *p*-value, and effect sizes as Hedges’ g (for mean differences) and rank-biserial r (for nonparametric contrasts) were reported. All within- and between-group *p*-values were corrected for multiple comparisons using the Benjamini-Hochberg false discovery rate (FDR) procedure, and the FDR-adjusted q-values are reported alongside the uncorrected *p*-values.

To assess potential attrition bias, comparisons between completers and non-completers were conducted both across the full sample (pooled analysis) and within each intervention group (Dance South, Nordic Walking, and Dance North), using the same parametric or non-parametric approach depending on distributional assumptions.

To further assess whether nominally significant between-group effects were supported at the participant level despite limited group sizes, we conducted an additional subject-level sensitivity analysis restricted to between-group contrasts that reached uncorrected *p* < .05 in the primary between-group change-score analyses (Tables S8 and S9). This restriction was applied to reduce multiplicity and to focus specifically on the subset of effects most relevant to between-group inference. For each such contrast, participants were classified according to whether their individual change score (Δ = post − pre) fell in the hypothesized beneficial direction for that metric, based on the *a priori* directional definitions described above (e.g., higher Granularity and HNR indicate improvement; lower Main Tone and Average pause duration indicate improvement). The proportion of participants showing beneficial change was then compared between groups using two-sided Fisher’s exact tests, given the small sample sizes. Analyses were performed for both task-level and composite contrasts. To assess whether the most robust between-group effects could be explained by key clinical characteristics, we conducted covariate analyses focused on the speech features that survived both the primary between-group analyses and the subject-level consistency analyses, namely Granularity and Main Tone at both the task and composite levels (Animal Fluency/Granularity, Routine/Main Tone, Composite/Granularity, and Composite/Main Tone). This restriction was applied to maintain consistency across analytic layers and to reduce multiplicity. We selected three covariates *a priori* on clinical grounds and to improve model stability given the final sample size: age, disease stage as indexed by the Hoehn and Yahr scale (H&Y), and global cognition as indexed by the Montreal Cognitive Assessment (MoCA). Age and H&Y were retained as core demographic and disease-severity variables, while MoCA was retained because the principal semantic outcome, Granularity, may plausibly depend on baseline cognitive status. Sex was not included in the adjusted models, since its inclusion as a covariate would yield unstable and unreliable estimates due to the sex imbalance across groups. For each retained outcome, multivariable general linear models (ANCOVA-style) were fitted with Δ score (post − pre) as the dependent variable and Group, Age, H&Y, and MoCA entered simultaneously as predictors. For each factor, we report the F-statistic, uncorrected *p*-value, FDR-corrected q-value, partial eta-squared (η^2^), and model R^2^ (Table S11). To examine the contribution of each covariate separately, we additionally fit a series of reduced models including Group and one covariate at a time. For these models, we report the F-statistic, *p*-value, FDR-corrected q-value, partial η^2^ for the covariate, and model R^2^ (Table S12). Finally, for the retained continuous covariates only (Age, H&Y, and MoCA), we summarized their unadjusted associations with Δ scores using simple linear regressions fitted separately for each retained speech outcome. For each regression, we report the slope (β), Pearson correlation coefficient (r), and *p*-value (Table S13). All analyses were conducted using two-sided tests.

### Additional resources

This study was conducted according to TREND (Transparent Reporting of Evaluations with Nonrandomized Design) Statement: Reporting of intervention evaluation studies using nonrandomized designs^92^ (DATA S2/Methods S2).

Clinical registry number: NCT06967493.

Full description of the protocol structure and session content: https://data.mendeley.com/datasets/3wgxpsbch7/1.

## Supplemental information

Document S1. Data S1/Method S1 and Data S2/Methods S2

Table S1. Baseline demographic and clinical characteristics comparing completers and non-completers participants

Table S2. Comparisons between completers and non-completers pooled across groups (overall attrition bias)

Table S3. Comparisons between completers and non-completers within group (group attrition bias)

Table S4. Individual pre-post data per participant, task, and speech metric

Table S5. Within-group pre-post analyses of speech metrics for animal fluency and routine tasks

Table S6. Individual composite pre-post data per participant and speech metric

Table S7. Within-group pre-post analyses of composite speech metrics

Table S8. Between-group comparisons of pre-post change (Δ) on speech metrics

Table S9. Between-group comparisons of pre-post change (Δ) on composite speech metrics

Table S10. Subject-level consistency analysis for between-group contrasts

Table S11. Multivariable models testing group and covariate effects on pre-post change (Δ)

Table S12. Single-predictor models testing the effect of individual covariates on pre-post change (Δ)

Table S13. Slopes of pre-post change (Δ) regressed on continuous covariates
